# The Value of Symmetry in Education and Character

**Published:** 1904-08

**Authors:** George F. Eames

**Affiliations:** Boston, Mass.


### THE VALUE OF SYMMETRY IN EDUCATION AND CHARACTER.[1]

BY GEORGE F. EAMES, M.D., BOSTON, MASS.

In the contemplation of any subject, be it animate or inanimate, the pleasure and satisfaction derived therefrom is dependent in a great degree on its symmetrical proportions; indeed, asymmetry, as seen in many objects, and especially in the human form, is a positive disfigurement, and is often repulsive.

But symmetry as I wish to discuss it will be considered in connection with the mind and soul. It will be noted at once that lack of symmetry in the mind is not so readily observed as a similar condition of the body, although they are vastly more common, and the deviations from what might be considered typical in the former would be altogether hideous if the same disproportion were exhibited in the physical system. Imagine a nose, which by a slight aberration of development has continued to grow until it is twelve inches long; or one eye eight inches in diameter and the other a mere pinhole! Such an extreme in physical deformity has probably never been known, yet similar disproportions, or greater, may easily be conceived to exist in the different powers of the mind. In the dental profession we should not expect to find excessive abnormalities or great deficiencies in intellect, but there are many indications that the majority of practitioners might de-

---

[1] Chairman's address before the Section on Stomatology at the fifty-fifth annual session of the American Medical Association, at Atlantic City, June 7 to 10, 1904.

velop their mechanical and intellectual powers in certain directions with great benefit to themselves and patients, and everlasting good to all people over whom they have any influence. This means that there are many defects in symmetry to be observed among dentists, and even among stomatologists.

While some defects may be found in the best of men, those which we have now under consideration are such that they, in many instances, ruin the man as a practitioner, and in nearly all the other cases mar his brilliancy and seriously handicap his professional progress. Take, for example, the life of the late P. D. Armour, the great capitalist of Chicago; his strict devotion to business, at the expense of all social life and entertainment, and his abstemious habits were still kept up even after his financial worth was counted by millions. While his business ability was increasing, other faculties of mind and heart were weakening, and when this hypertrophic condition, in the shape of an enormously enlarged business capacity, became pathologic, and the discrepancy between this and other bodily and mental functions became so great that he began to suffer from the strain, he was urged to rest and go to Europe. He went, but he had lost all capacity for the enjoyment of travel and change of scene, and he died in the harness which he had himself constructed. The following quotation from Charles Robert Darwin shows still further the effect of asymmetrical development:

"I have said that in one respect my mind has changed during the last twenty or thirty years. Up to the age of thirty, or beyond it, poetry of many kinds, such as the work of Milton, Gray, Byron, Wordsworth, Coleridge, and Shelly, gave me great pleasure, and even as a school-boy I took great delight in Shakespeare, especially in the historical plays. I have also said that formerly pictures gave me considerable pleasure, and music very great delight. But now for many years I can not endure to read a line of poetry. I have tried lately to read Shakespeare, and found it so intolerably dull that it nauseated me. I have also almost lost my taste for pictures or music. . . . This curious and lamentable loss of the higher æsthetic tastes is all the odder, as books on history, biographies and travels (independently of any scientific facts which they may contain), and essays on all sorts of subjects, interest me as much as ever they did. . . . Why this should have caused the atrophy of that part of the brain alone on which the higher tastes depend I can not conceive. . . . If I had to live my life over again, I would make it a rule to read some poetry and listen to some music at least once every week, for perhaps the parts of my brain now atrophied would have thus been kept active through use. The loss of these tastes is a loss of happiness, and may possibly be injurious to the intellect, and more probably to the moral character, by enfeebling the emotional part of our nature."

Professor Charles Eliot Norton says, "Whatever your occupation may be, and however crowded your hours with affairs, do not fail to secure at least a few minutes every day for refreshment of your inner life with a bit of poetry."

A few weeks ago I met a physician in the suburbs of Boston. He was apparently taking a walk for pleasure, and was observing some birds by the way. My first thought was one of surprise that this physician, in full practice, the author of a large work and in many ways one who must meet numerous demands, could and would find time for such a purpose, but a moment's reflection convinced me that such use of time was very wise and judicious—that it was necessary, not alone for diversion and recreation, but that it served to cultivate the mind, to broaden the view, and to render more sensitive and acute the artistic and moral sense. In many other ways a study of the lower animals increases, by comparison, our knowledge of the higher; and this is, as we well know, of essential value to the physician. It is a grave mistake to neglect the cultivation of the artistic and æsthetic sides of our nature, of a love of the beautiful, of sympathetic feelings and of all that is delicate and refined, whether one is fitting to become a physician, a general, or a special surgeon.

Away with the idea that the surgeon needs no feelings of delicacy and sympathy; that, devoid of all sense of feeling, he cuts across nerve and artery, or that he administers an anæsthetic which suspends nearly all the functions of life without giving a thought to the real condition in which he places his patient—without feeling the full weight of responsibility in holding a precious life in his hands! It is true that a surgeon should not allow his feelings—his emotions—to be uppermost when he is performing an operation. To all appearance he must be devoid of sensibility, and even sympathy—yet the world knows that the greatest delicacy of perception, the keenest appreciation of possible pain are a part of his equipment, and that it is only the well-being of the patient which demands apparent indifference to suffering. If he can not, for the time at least, lock up his "feelings" so that they will disturb neither his patient nor himself, he loses something of his dexterity and physical poise. But sensitiveness—mental as well as physical—is a very important characteristic of a great physician, and this same sensitiveness is cultivated or lessened, as the case may be, by a man's daily living and his attitude towards life. He may harden and blunt his perceptions as he may, dull his tools if he will, but good work demands the keenest edge of which he is capable.

The practical and important issue before us, in view of the conditions to which reference has just been made, is this: What are the more important defects in our professional life, and what are the remedies? The first consideration in seeking to correct a diseased or any undesirable condition is to discover the cause, and here it is to be sought in the early years of child-life, although, indeed, causes may exist generations before. Manifestly, the suitable preparation of body and mind for the duties of life may be included in the broad term, education. Asymmetry due to lack of development, then, of the body or mind is due to faulty education. Many of our college graduates are like statues, carved to be placed in a frieze. The side towards the public gaze defies criticism, but the rest is of rough, unhewn stone.

It is evident that life is too short to pursue studies in every direction available at the present time, therefore a choice must be made; on this choice may hang future success or failure, and to it we may look for the cause of many deficiencies in development of both body and mind. The writer believes that much is taught in all departments of education which has no vital and practical value in the life work to come. With innumerable subjects for study, with the many subdivisions of these subjects and the almost unlimited extent to which they may be pursued, the great need is shown of weeding out, and also the serious difficulty of doing so. Everything which is taught at the present time has a certain value, and one reluctantly strikes out of the list anything which is worth while, but it is a choice between greater and lesser values. From early childhood it should become a matter for serious consideration, not only as to what shall be taught, but also as to what needs curbing, and what should be strengthened. Mere intellect, as some one has said, is as hard-hearted and as heart hardening as mere sense. We need to remember this in our schemes of education. A

little child, instead of being confined in a room for hours, where he is taught the alphabet or spelling, would be far better off were he out in the fresh air and sunlight, becoming familiar with the colors and flowers and the facts of nature.

Later in life, in the preparatory schools, no one will question that mathematics is an essential item in the curriculum, yet even here we may make an improvement in the old methods by substituting something more useful and practical for the higher mathematics, leaving these until later, or until the life work is chosen.

The writer has in his school-days spent much time over mathematical problems or catch-traps which had no value beyond the discipline and exercise which it gave the mind, and he believes that something else might have been substituted which would have given the necessary discipline and exercise, and at the same time would have educated me in a useful and practical way. On the other hand, overindulgent parents and unthinking teachers may allow a child to follow his bent too closely, and the very danger is precipitated which we wish to avoid. In order to produce a symmetrical figure, the pedestal or foundation must be strong and well rounded, so that however elaborate the decoration of the capital or head of the column may be, there shall be no incongruity between it and its base. A school course which should fitly train mind, heart and body in the right degree would be the ideal one.

It would seem to be a wise procedure if exercises were introduced at an early age with the object of ascertaining the prominent characteristics inherent in each child; for instance, even in kindergarten life, a talent, or the ability to do one thing better than another, is often shown, as are the inventive and constructive powers. Equal attention should, of course, be given to finding out and strengthening weak points. This, if systematically carried out during school life, and a record made and kept for future reference, would be valuable. For example, a young man, with the help of his friends, wishes to determine in what direction his life work shall be. In coming to this decision he not only considers what he thinks he would like to do, what he is most interested in, and how he could obtain the most money, but he should by no means neglect to take into consideration the direction in which his greatest talents lie, the things which he has done with the most marked ability, the work which by nature he is best adapted to do. For this purpose recourse may be had to his school record, which is on file at the schools which he has attended. This record will show, from child life up, the individual's proficiency in drawing and other work which shows artistic ability, also the student's aptness in a mechanical and manipulative way. The inventive powers are often shown even in kindergarten life in the various shapes and ways in which blocks may be arranged, etc.

These and other items of the student's school record should be of great assistance in the choice of a vocation. To take up the life work to which one is best adapted, means the greatest possible usefulness to mankind, the happiest and most harmonious life and the most symmetrical growth. Again, this system of recording should be of great service to those who have charge of the entrance of candidates into colleges of dentistry, law, medicine, and others. This record should stand not only for the applicant's ability as a student, but for his special adaptability to a given profession, and no other entrance examination should be needed. Why should any second examination be made if the first is satisfactory? Is it not a declaration that the first is not to be depended on if the record is made? What a comment on the learning and teaching ability of our esteemed professors in the dental colleges, that their graduates with a diploma bearing their signature and the seal of the college should fail to satisfy a State board of examiners, or be debarred practice in another State! Surely, there is a great lack of symmetry in the results of our modern college work; if not, then the work of the state board of examiners is unnecessary, for no one should be allowed to practise as a stomatologist without a diploma, and that diploma should stand as the best passport to practice. Regarding the standard of entrance to colleges of stomatology, it should undoubtedly be high, but even this may be overdone and ill-advised. To the writer's knowledge, many who have entered the dental college as graduates of a high school have been among the worst specimens of graduate practitioners, and far below in quality those who had been received without having had such a course. Graduation from a high school as a minimum standard may be used as a basis, but this alone is not sufficient. If symmetry be wanting, the high school graduate is of poor promise and his entrance to the dental college should be postponed or discouraged. If a degree is required for entrance, and it undoubtedly should be, still it is necessary to look for other qualifications, especially if degrees may still be obtained from some sources for money and while it shows nothing of the candidate's manipulative dexterity.

By lack of symmetry in such cases is meant a deficiency in mechanical ability or literary culture, for instance, to such an extent that it would be unwise to receive the candidate. On the other hand, a young student who, for some good reason, has not graduated from the high school, may have such a symmetrical record as far as he has gone, and shows such qualifications of promise, that it would be very unwise to reject him. Of course, desirable candidates do not need an entrance examination; they do not need a four years' course or one of two years; they would reach the high places in the profession in spite of the entrance examination and the college courses, and they are so well endowed, mentally and physically, that they are able to decide as to their own fitness for a certain profession. Their own desire and ambition is for the best things, and they are not limited by years in a college; they would even get on without the college, and no ordinary hinderance could prevent their success. A friend of mine, a physician, now one of my listeners, was with me a few years ago in the Tower of London, and we stood on the spot where Anne Boleyn, Lady Jane Grey, and Katherine Howard were beheaded. My friend the doctor remarked, "If they'd let them alone they would have died themselves long ago." So with the most desirable men of our profession; they are such that if we let them alone they will go on with their professional development without regulations as to entrance standards and length of college courses that we spend so much time in talking about. The problem is to keep out incompetents, and the unworthy, even if they may have in a fashion "gone through" some school or college with the required standard. We might have a ten-year course for Jack Blunthead, and he would still be unfit, while Joseph Sharp has passed all the requirements of the ordinary graduate in two years. A matter of years alone, or even the administration of thyroid extract, will not always qualify, and there is nothing that will qualify if there be not inherent in the individual the incentive and the genius to succeed. Quality, not quantity, is the essential requirement, and if this be attained, what can it matter whether the student has achieved it in one year or four, although the preference should be given to the one-year man? Therefore, when it is proposed to extend the dental course to four years, I am opposed to it; but this should not, by any means, convey the impression that anything but the highest standard is advocated.

Time is too precious to compel such men as we have pronounced worthy to enter the dental college, to give four years of their life in it. Three years is enough time to give to that part of the student life which is spent within the college walls. To all right-minded graduates, the end of the college course is but the beginning of studentship in earnest. Out of college the student, now also a practitioner, has entire management of his education and the development of his talents, and his success and future degree of symmetry, the nearness with which he comes to the finished product in man, is limited only by himself.

Among practitioners many defects in symmetry may be observed. There are some practitioners who have developed their mechanical powers to such an extent, and have become so absorbed in some mechanical specialties, so-called, that they are unable when they look at the human mouth to see anything but a possible bridge, crown, or plate; others have a clear vision as to dollars, their other sense perceptions being weakened from disuse. In fact, atrophy is often associated with hypertrophy, and the former may be an indirect result of the latter, both physically and mentally.

Specialties and specialists have done much to advance their particular lines of work, and this is greatly to be commended, but there are disadvantages as well, and these consist in the neglect of general considerations, which, if given some attention, would help much towards a more symmetrical condition.

In the enthusiasm and interest which is taken in certain lines of work in our profession, many of us forget that there are other departments of our work which should also receive attention; that, in many directions, our mental and bodily capacity is weakening through loss of function; that we are better dentists by being better citizens; that we are the best stomatologists when we have cultivated our hearts, minds, and bodies in all possible directions around the one central object which draws us all together with a common interest and a common purpose.

